# Knowledge, Attitudes, and Practices Related to Cholera Outbreak Among Medical Students in Yemen: A Cross-Sectional Study

**DOI:** 10.7759/cureus.78885

**Published:** 2025-02-12

**Authors:** Shahd Alqato, Hebat Allah N Fadhl, Naji Al-Bawah, Ayah Abdulgadir, Houssaini Mohamed Amine, Ruqia H Alsomali, Mazin Taha, Naif Abduljabbar, Sarah Al-Fadhel, Mohammed A Saghir

**Affiliations:** 1 Internal Medicine, Arab Medical Center, Amman, JOR; 2 Medical Education, University of Aden, Aden, YEM; 3 Medicine, Sana'a University, Sana'a, YEM; 4 Medicine, University of Khartoum, Khartoum, SDN; 5 General Practice, Faculty of Medicine and Pharmacy of Oujda, Oujda, MAR; 6 Nursing, King Fahad General Hospital, Jeddah, SAU; 7 Surgery, Al-Fajr College of Science and Technology, Khartoum, SDN; 8 Community Health Nursing, University of Khartoum, Khartoum, SDN; 9 Internal Medicine, 21 September University, Sana'a, YEM; 10 Graduate College, University of Bahri, Khartoum, SDN; 11 Medicine, Cairo University, Cairo, EGY

**Keywords:** acute diarrheal disease, cholera outbreak, civil war, undergraduate medical student, yemen

## Abstract

Background

Cholera is a potentially life-threatening diarrheal disease if left untreated, transmitted via contaminated water or food, and linked mainly to poor sanitation. Yemen is facing a public health crisis with an increasing number of cases in 2024, underscoring the importance of improving awareness and the need for education to enhance prevention and management.

Methods

This observational cross-sectional study was conducted in July 2024. The data were collected via a self-administered online questionnaire. Inferential analysis included independent t-tests for comparing two groups and one-way ANOVA for multiple groups, with a statistical significance threshold of p<0.05.

Results

The study surveyed 412 Yemeni medical students, predominantly male (65.5%) and single (86.7%), with a mean age of 22.48±2.6 years. The mean knowledge score was 7.35±2.36, and significant knowledge gaps in recognizing the range of severity of the disease, risk factors, complications, and vaccine awareness exist. The mean attitude score was 11.45±2.13 out of 15, with 70% showing a positive attitude. The practices score was poor, with a mean of 4.32±2.18 out of 9, with low adherence to preventive measures such as washing and peeling fresh fruits and vegetables (<30%). Our analysis showed significant associations, with age positively correlating with knowledge (r=0.262; p<0.001) and attitudes (r=0.17; p<0.001), while females scored higher in practices (p=0.002). Academic year significantly influenced knowledge (p<0.001) and attitudes (p=0.003), with sixth-year students scoring the highest.

Conclusion

While the Yemeni medical students demonstrated moderate knowledge and a generally positive attitude toward cholera, significant gaps in preventive practices were noted. Addressing these gaps through targeted educational programs on disease prevention, transmission, and management is essential to strengthen students' preparedness and improve public health outcomes.

## Introduction

Cholera is a potentially life-threatening diarrheal disease caused by infection by the bacteria *Vibrio cholerae*, with epidemic and pandemic tendencies, as the two serogroups, O1 and O139, of bacteria that survive in conditions with poor sanitation have been associated with outbreaks worldwide and they produce a potent enterotoxin (cholera toxin) responsible for the lethal symptoms of the disease [1]. The disease is transmitted through two main routes, primary transmission from aquatic environments and secondary transmission from infected individuals, with the latter driving outbreaks and epidemics in endemic areas. Contaminated water is the primary vehicle in endemic regions, while primary transmission is often derived from food, such as undercooked seafood. The infective dose depends on individual susceptibility, with higher doses required for healthy individuals and lower doses affecting those with reduced gastric acid protection or poor health [1]. The disease often presents with painless, rice-water stools and varying dehydration severity, with mild or asymptomatic cases being the majority; however, severe cases can lead to rapid fatality within hours if untreated. Prompt fluid and electrolyte replacement therapy can reduce mortality to as low as 1% [2]. This disease is currently a global health concern, particularly in regions with limited access to safe drinking water and adequate sanitation infrastructure. According to the World Health Organization, the disease incidence is estimated to be between 1.3 and 4 million new cases of cholera and 21,000 and 143,000 deaths worldwide each year [2]. Cholera is endemic in approximately 50 nations, largely throughout Asia and Africa [3]. Several historical epidemics have been reported, with the most recent occurring in 2022 in Lebanon, which is linked to the spread from Syria, thousands of cases, and several deaths [4]. In Yemen, the disease remains a severe, persistent public health crisis due to ongoing conflicts in the area and inadequate sanitation measures. From 2016 to 2022, Yemen recorded the largest cholera outbreak in recorded history, with over 2.5 million suspected cases and 4,000 deaths. Experts predict that without scaling up crisis management plans, the number of cases in 2024 could increase to more than a quarter of a million by September [5].

The knowledge, attitudes, and practices (KAP) of medical students regarding cholera reflect not only the effectiveness of their education but also their potential to raise awareness and enhance community health as future healthcare providers and current advocates for health. In our study, we aimed to understand how medical students in Yemen perceive and approach cholera and explore the factors that shape their understanding, with the goal of suggesting strategies to improve disease prevention and outbreak management efforts.

## Materials and methods

Study design and study population

This observational cross-sectional study was conducted in July 2024 after obtaining approval from the Departmental Research Unit of Sana'a University (approval number: 1325). The study targeted Doctor of Medicine students in Yemen aged 18 years or older. Students younger than 18 years, those who did not complete the questionnaire, or those who declined participation were excluded. Participation was voluntary, and electronic informed consent was obtained from all the respondents. The study adhered to the Strengthening the Reporting of Observational Studies in Epidemiology (STROBE) guidelines, as outlined by the Enhancing the Quality and Transparency of Health Research (EQUATOR) network [6].

Study collection tool

Data were collected by a valid online self-administered questionnaire (Appendices). The survey questions were primarily in English and consisted of multiple-choice and closed-ended choices, with some items allowing the selection of multiple answers. The questionnaire was developed on the basis of a literature review, with most items adapted from a study conducted in Syria that explored a similar aspect [7]. The data were divided into four sections, beginning with an introduction highlighting the study objectives and assuring the confidentiality of the collected information, followed by a mandatory electronic consent item. The first section covered demographic information, consisting of five items, including variables such as age, sex, and year of study. The second section was the knowledge scale, which included 15 items assessing their understanding of its causative agent, transmission, symptoms, prevention, and treatment. The third section explored students' attitudes toward the disease via three questions on a 5-point Likert scale: a question on how dangerous the disease is perceived to be (5 indicating "extremely dangerous"), the level of concern about its re-emergence (5 representing "extremely concerned"), and the degree of personal worry about becoming infected (5 reflecting "extreme worry"). The final section evaluated students' practices toward cholera prevention via a scale of 9 points, with points assigned for behaviors considered effective in reducing the risk of cholera infection. The first question addressed eating habits, with eating out or ordering takeaway fewer than five times per month classified as a good practice, earning a point. The second and third questions focused on handling raw vegetables and fruits, where the ideal practice of washing and peeling them was awarded a point each. The final question introduced six preventive measures in response to the re-emergence of cholera, with points awarded for each practice, including washing fruits and vegetables more thoroughly, paying attention to the sources of drinking water, and others. The questionnaire was previously tested for clarity, accuracy, and face validity by previous researchers from a similar study in Syria [7]. We calculated Cronbach's alpha for each scale to assess internal consistency, yielding the following results: knowledge scale (α=0.70), practice scale (α=0.70), and attitude scale (α=0.74). The values were deemed accepted by us, given the exploratory nature of our research.

Sample size and collection

The sample size was calculated via the Cochran formula (n=z²p(1-p)/d²) [8], with the following key parameters: a confidence level of 95% (corresponding to a Z-score of 1.96), a margin of error of 5% (E=0.05), and an assumed prevalence of 50% (P=0.5), since the number of medical students in Yemen has not been reported before. The minimum required number of participants was 385. Responses were collected via a self-administered online questionnaire uploaded electronically on Google Forms. It was distributed through social media platforms using the convenience sampling method, starting with official medical student social media channels in Yemen. Four hundred and twelve medical students responded to the survey invitation and completed it.

Statistical analysis

The data analysis was done via IBM SPSS Statistics for Windows, Version 28.0 (Released 2021; IBM Corp., Armonk, New York, United States) [9]. We presented continuous variables using measures of central tendency, such as the mean and standard deviation, and categorical variables (e.g., gender) using frequency tables. For inferential analysis, the independent t-test was used to compare mean differences between two groups, whereas one-way ANOVA was used to compare means across multiple groups. A p-value of less than 0.05 was considered statistically significant. Descriptive and analytic statistics were used to summarize demographic characteristics, knowledge questions, and other dependent variables in the tables. The knowledge score was calculated by summing the scores for individual items, with correct responses assigned a value of 1 and incorrect or "I do not know" answers assigned a value of 0, with a maximum score of 15. Attitudes were evaluated on a scale ranging from 3 to 15, with higher scores indicating a more positive attitude. Practices were assessed similarly and scored on a scale from 0 to 9, where higher scores reflected more cautious practices. We further classified commitment to protective practices levels based on the total score for the fourth question: a score of 0-3 indicated poor commitment, while a score of 4-6 represented good commitment.

## Results

Characteristics of the population and descriptive statistics

Our study included 412 medical students from Yemen; most of them were males (n=270; 65.5%) and single (n=357; 86.7%), and their mean age was 22.8±2.7 years. Fifth-year medical students composed more than a quarter of the sample (n=109; 26.5%), and the majority of participants resided in Sana'a (n=271; 65.8%), as demonstrated in Table 1.

**Table 1:** Sociodemographic characteristics (n=412) n: frequency; %: percentage; SD: standard deviation

Characteristics	N	%
Age (mean, SD)	(22.4, 2.77)	
Gender		
Male	270	65.5
Female	142	34.5
Marital status		
Single	356	86.7
Married	56	12.9
University		
Sana'a University	271	65.8
Others	141	34.2
Academic year		
1st year	63	15.3
2nd year	56	13.6
3rd year	78	18.9
4th year	72	17.5
5th year	109	26.5
6th year	31	7.5

Knowledge score on cholera

The mean knowledge score was 7.35±2.36 out of 15, indicating inadequate knowledge levels among the students. Most students correctly identified the infectious agent as a bacterium, the oral route of transmission as the primary entry point to the body, the vulnerable groups, rehydration as the main treatment, and the digestive system as the primarily affected system (359 (87.1%), 371 (90%), 392 (95.1%), 335 (81.3%), and 380 (92.2%), respectively). However, significant gaps were noted among students in recognizing clinical manifestations, the range of disease severity, incubation period, risk factors, possible complications, vaccine availability, and other preventive measures (80 (19.4%), 7 (1.7%), 137 (33.3%), 20 (4.9%), 101 (24.5%), 148 (35.9%), and 80 (19.4%), respectively). The detailed percentages and numbers of questions answered correctly from the knowledge scale are illustrated in Table 2.

**Table 2:** Knowledge items regarding cholera (n=412) n: frequency; %: percentage; SD: standard deviation

Question	Correct answer, n (%)
What type of infectious agent causes cholera?	359 (87.1)
Through which routes does the cholera pathogen enter the human body?	371 (90)
What are the modes of transmission for cholera?	51 (12.4)
What are the clinical manifestations of cholera infection?	80 (19.4)
How would you classify the severity of symptoms in cholera cases?	7 (1.7)
What is the typical incubation period for cholera?	137 (33.3)
What are the risk factors that increase susceptibility to cholera infection?	20 (4.9)
What are the potential complications associated with untreated cholera?	101 (24.5)
Which age groups are most vulnerable to cholera infection?	392 (95.1)
What are the main treatments?	335 (81.3)
Is there a vaccine available for cholera?	148 (35.9)
Can cholera be transmitted between humans and other species?	313 (76)
How is a diagnosis of cholera confirmed?	327 (79.4)
Which body systems are primarily affected by cholera?	380 (92.2)
What are the preventive measures to reduce the risk of cholera infection?	80 (19.4)
Total mean score±SD	7.35±2.36

Attitudes toward the cholera outbreak

The majority of students (n=290; 70%) achieved high attitude scores, with a mean of 11.45±2.13, reflecting students' positive attitudes toward the disease. About 84.5% of students perceived cholera as either very dangerous (164, 39.8%) or extremely dangerous (184, 44.7%). Moreover, more than two-thirds of students were very concerned (138, 33.5%) or extremely concerned (148, 35.9%) about cholera re-emerging in the country. Only a small percentage were slightly concerned (33, 8%) or not at all concerned (8, 1.9%). Despite acknowledging that cholera is a serious disease, fewer than half of the students were very worried (89, 21.6%) or extremely worried (106, 25.7%) about contracting the disease themselves, as illustrated in detail in Table 3.

**Table 3:** Frequency and percentages of medical students' answers on the attitude scale (n=412) n: frequency; %: percentage

Attitude aspect	Scale				
How dangerous do you perceive cholera to be?	Not at all dangerous n (%)	Slightly dangerous n (%)	Moderately dangerous n (%)	Very dangerous n (%)	Extremely dangerous n (%)
	6 (1.5)	20 (4.9)	38 (9.2)	164 (39.8)	184 (44.7)
How concerned are you about cholera re-emerging in the country?	Not at all concerned n (%)	Slightly concerned n (%)	Moderately concerned n (%)	Very concerned n (%)	Extremely concerned n (%)
	8 (1.9)	33 (8)	85 (20.6)	138 (33.5)	148 (35.9)
How worried are you about getting cholera infection?	Not at all worried n (%)	Slightly worried n (%)	Moderately worried n (%)	Very worried n (%)	Extremely worried n (%)
	40 (9.7)	58 (14.1)	119 (28.9)	89 (21.6)	106 (25.7)

Practices toward cholera outbreaks

Almost half of the students (n=220; 53.4%) had poor practices scores, with a mean of 4.32±2.18 out of 9. The practice of eating in restaurants out of home per month was variable, with 38.1% of the students eating fewer than five times per month outside and 22.6% eating more than 20 times per month. Fewer than 30% of the students consistently washed and peeled raw or fresh vegetables (n=118; 28.6%) or fruits (n=121; 29.4%), as shown in detail in Table 4. A total of 179 (43.4%) of the students showed poor commitment to changing behaviors related to cholera after the re-emergence of the disease, whereas the remaining students showed a good level of commitment to changing behavior.

**Table 4:** Practice toward cholera among the students (n=412) n: frequency; %: percentage

Practice	Answer	n (%)
How often do you eat out or order takeaway in a typical month?	<5*	157 (38.1)
	5-10	99 (24)
	10-20	63 (15.3)
	>20	93 (22.8)
What do you usually do before consuming raw vegetables?	Wash (water only)	255 (61.9)
	Wash and peel*	118 (28.6)
	Do not wash it nor peel it	39 (9.3)
What steps do you take before eating fresh fruits?	Wash (water only)	274 (66.5)
	Wash and peel*	121 (29.4)
	Do not wash it nor peel it	17 (4.1)
After the newest re-emergence of cholera, you commit to:	Wash fruits and vegetables more thoroughly*	331 (80.3)
	Wash hands with soap more frequently*	326 (79.1)
	Pay more attention to sources of drinking water*	280 (68)
	Minimize eating out/takeaway*	233 (56.6)
	Avoid eating undercooked seafood and sushi*	153 (37.1)
	Do not eat unpeeled fresh produce*	99 (24)
	Not changing my practices	35 (8.5)

Inferential statistics

Our analysis revealed significant positive associations between sociodemographic characteristics and KAP scores. Age was positively correlated with both knowledge (r=0.262; p<0.001) and attitudes (r=0.17; p<0.001). Married participants scored higher in knowledge (8.08±1.92) compared to single participants (7.47±2.07; p=0.047). Female students scored higher in practices (4.85±2.05) compared to males (4.19±2.08; p=0.002). A one-way ANOVA test revealed a significant positive trend in knowledge (p<0.001) and attitude (p=0.003) scores across academic years, with higher scores observed in upper years, particularly among sixth-year students (knowledge: 8.58±2.23; attitudes: 11.90±1.99). Post hoc analysis showed significant differences in knowledge scores between second and third years (MD: -1.400; p=0.001), second and fourth years (MD: -1.070; p=0.021), and second and sixth years (MD: -2.136; p<0.001). Attitude scores also improved, with significant differences between second and third years (MD: -1.269; p=0.042) and second and fourth years (MD: -1.597; p=0.003). Detailed correlations are illustrated in Table 5 and Table 6.

**Table 5:** Relationships between knowledge, attitudes, and practices scores and sociodemographic characteristics (n=412) Data is reported as mean±SD. * indicates statistical significance at p<0.05 r: Pearson correlation coefficient; ^a^:Pearson correlation test; ^b^: independent sample t-test; ^c^: one-way ANOVA (Welch's test)

Variable	Knowledge score	P-value	Test value	Attitude score	P-value	Test value	Practices score	P-value	Test value
Age^a^	-	<0.001	r=0.262	-	<0.001	r=0.17	-	0.636	r=0.024
Gender^b^									
Male	7.59±2.13	0.615	T-value=0.504	11.37±2.5	0.076	T-value=-1.780	4.19±2.08	0.002*	T-value=-3.077
Female	7.48±1.91			11.82±2.27			4.85±2.05		
Marital status^b^									
Single	7.47±2.07	0.047*	T-value=-1.992	11.48±2.42	0.399	T-value=-0.8452	4.41±2.04	0.962	T-value=0.0480
Married	8.08±1.92			11.78±2.45			4.4±2.40		
University^b^									
Sana'a University	7.61±2.1	0.464	T-value=0.733	11.56±2.5	0.703	T-value=0.381	4.49±2.06	0.271	T-value=1.102
Other universities	7.45±1.97			11.46±2.29			4.26±2.15		
Academic year^c^									
1st year	6.98±2.06	<0.001*	F-value=6.368	11.03±2.52	0.003*	F-value=0.744	4.22±2.29	0.848	F-value=0.401
2nd year	6.44±2.03			10.5±2.54			4.48±2.11		
3rd year	7.84±1.84			11.77±2.31			4.62±2.03		
4th year	7.51±1.51			12.1±2.06			4.24±1.96		
5th year	7.92±2.15			11.63±2.58			4.36±2.09		
6th year	8.58±2.23			11.90±1.99			4.55±2.16		

**Table 6:** Post hoc comparisons of knowledge, attitudes, and practices scores by academic year using the Games-Howell test (n=412) * indicates statistical significance at p<0.05

Comparison	Knowledge		Attitudes	
	Mean difference	P-value	Mean difference	P-value
1st vs 2nd year	0.539	0.717	0.532	0.862
1st vs 3rd year	-0.860	0.114	-0.737	0.474
1st vs 4th year	-0.531	0.560	-1.065	0.090
1st vs 5th year	-0.9407	0.062	-0.601	0.669
1st vs 6th year	-1.597	0.018*	-0.871	0.458
2nd vs 3rd year	-1.400	0.001*	-1.269	0.042*
2nd vs 4th year	-1.070	0.021*	-1.597	0.003*
2nd vs 5th year	-1.4801	-	-1.133	0.083
2nd vs 6th year	-2.136	-	-1.403	0.061
3rd vs 4th year	0.329	0.845	-0.328	0.941
3rd vs 5th year	-0.0804	1.000	0.136	0.999
3rd vs 6th year	-0.736	0.585	-0.134	1.000
4th vs 5th year	-0.4098	0.681	0.464	0.764
4th vs 6th year	-1.066	0.173	0.194	0.998
5th vs 6th year	-0.656	0.696	-0.270	0.989

The regression analysis revealed significant predictors of KAP. For knowledge, respondents in Years 3, 5, and 6 exhibited significantly higher scores compared to Year 1, with the largest increase observed in Year 6 (β=1.610; 95% CI: 0.737-2.482; p<0.001). Regarding attitude, Year 4 respondents displayed significantly higher scores compared to Year 1 (β=0.993; 95% CI: 0.165-1.820; p=0.019). For practices, females demonstrated significantly better adherence than males, as reflected by the negative association for females (β=-0.614; 95% CI: -1.047 to -0.181; p=0.006), as shown in Table 7.

**Table 7:** Linear regression analysis of possible predictors of knowledge, attitudes, and practices related to cholera among the students (n=412) * indicates statistical significance at p<0.05

Predictor	Knowledge			Attitudes			Practices		
	β	95% CI	P-value	β	95% CI	P-value	β	95% CI	P-value
City (non-Sana'a vs. Sana'a)	-0.100	(-0.545, 0.346)	0.661	-0.058	(-0.592, 0.477)	0.832	-0.323	(-0.788, 0.141)	0.172
Year (2-1)	-0.480	(-1.233, 0.273)	0.211	-0.589	(-1.491, 0.314)	0.201	0.290	(-0.494, 1.075)	0.468
Year (3-1)	0.937	(0.245, 1.628)	0.008*	0.714	(-0.120, 1.548)	0.093	0.447	(-0.279, 1.172)	0.227
Year (4-1)	0.579	(-0.112, 1.271)	0.100	0.993	(0.165, 1.820)	0.019*	-0.045	(-0.765, 0.674)	0.902
Year (5-1)	0.964	(0.341, 1.586)	0.002*	0.537	(-0.211, 1.286)	0.159	0.042	(-0.608, 0.693)	0.899
Year (6-1)	1.610	(0.737, 2.482)	-	0.771	(-0.285, 1.827)	0.152	0.290	(-0.628, 1.209)	0.534
Gender (male-female)	0.198	(-0.218, 0.614)	0.350	-0.360	(-0.858, 0.138)	0.156	-0.614	(-1.047, -0.181)	0.006*
Marital (married-single)	0.567	(-0.006, 1.140)	0.052	0.319	(-0.364, 1.003)	0.359	0.001*	(-0.593, 0.596)	0.996

In line with these findings, Spearman correlation coefficients further revealed significant relationships between the three scores, with knowledge and attitude scores showing a weak positive correlation (r=0.247; p<0.001), knowledge and practices scores showing a strong positive correlation (r=0.261; p<0.001), and attitude and practices scores showing a moderate positive correlation (r=0.263; p<0.001).

## Discussion

This study evaluated KAP toward an emerging outbreak of *Vibrio cholerae* in Yemen among medical students. Cholera remains a critical public health challenge in Yemen, further affected by the ongoing conflict and inadequate sanitation in the country [5]. As of September 2024, the International Organization for Migration warned that the number of cases could exceed 250,000 without effective interventions. This issue is not new, as Yemen experienced the largest documented cholera outbreak between 2016 and 2022, with over 2.5 million cases. Addressing healthcare workers' knowledge and preparedness is crucial for controlling this disease. Therefore, in this study, we aimed to evaluate Yemeni medical students' KAP regarding cholera [5]. A total of 412 medical students from various universities in Yemen participated in the survey. Our analysis revealed that while most students exhibited moderate knowledge (mean score of 7.35±2.36), significant gaps were identified in their understanding of prevention and risk factors. Despite demonstrating a positive attitude toward cholera control (mean score of 11.45±2.13), their practical implementation of preventive measures was notably inadequate, with a significantly low mean score of 4.32±2.18. Moreover, advancement in study years has significantly increased knowledge and attitude scores. These findings provide insights for curriculum developers in Yemen and other developing countries facing the pandemic to enhance education on cholera. The incorporation of targeted content on infection risk, transmission routes, preventive measures, and management into the curriculum can help equip students to address the disease more effectively and promote public health awareness.

Despite students demonstrating moderate knowledge about cholera, we noticed significant gaps in identifying all infection sources and risk factors (only 4.9% identified all basic risks, including poor sanitation and hygiene). Awareness of the disease's clinical severity spectrum was especially low, with under 2% recognizing its range from asymptomatic to severe. Preventive behavior awareness was also inadequate, with fewer than 20% identifying all basic measures, such as drinking clean water (bottled or previously boiled) or washing hands with soap well and frequently. Two previous studies among the Yemeni population in Aden [10] and Al-Mahweet [11] and a study from Tanzania [12] revealed similar gaps in awareness about transmission routes and prevention methods. Our findings shed light on a concerning matter, as students who received medical education do not have adequate awareness of those aspects and are relatively similar to the general population, indicating an area of improvement in the educational curriculum of medical students in Yemen. Although the efficacy of cholera vaccination in outbreak control remains under scientific discussion, it is widely recognized as a critical tool for protecting high-risk individuals in endemic regions [13]. In our study, only 35.9% of the students were aware of the vaccine's existence. Conversely, a study from Lebanon revealed higher levels of public knowledge regarding cholera prevention measures, including awareness of the cholera vaccine, with approximately twice the level of awareness reported among Yemeni students, possibly due to the extensive media coverage and governmental initiatives regarding the disease with the neighboring counties' outbreaks [14]. Similar significant correlations between KAP aspects were reported in other studies in Kenya [15] and Lebanon [4], emphasizing how educational interventions that enhance knowledge can improve attitudes and positively shape public health behaviors.

The good attitude score aligns with findings from a similar study in Aden, Yemen, among the general population, indicating that the population is cautious about the disease [10], likely driven by Yemen's frequent outbreaks and vulnerability due to limited healthcare and sanitation resources. Conversely, a study conducted in Jazan, Saudi Arabia, a close geographical country with a more robust socioeconomic profile, revealed that more than half of the participants had poor attitudes toward preventive measures against cholera [16].

Despite medical students' positive attitudes, they exhibited low levels of protective practices. This could be explained by the fact that a positive attitude alone may not necessarily correlate with improved practices among healthcare workers [17]. Additionally, studies have noted that while low socioeconomic status is associated with a more cautious attitude toward cholera, it did not correlate with better preventive practices in a study from Ghana, possibly because of inadequate water and sanitation infrastructure rather than a personal unwillingness to adopt the best practices [18]. The findings from the study in Saudi Arabia [16] were similar in terms of poor sanitation practices, potentially influenced by the cultural characteristics shared between the two countries. An alarming observation is that less than 30% of participants committed to washing and peeling fresh fruits or raw vegetables before consumption, as inadequate washing of fruits or vegetables increases the exposure to different kinds of pathogens, resulting in a high prevalence of waterborne outbreaks, including cholera [19], further indicating a lack of awareness regarding cholera transmission routes and suggesting significant gaps in sanitary practices. In comparison, a study from Syria, another country affected by conflict and economic hardship, showed that over 80% of participants adhered to this practice [7], highlighting a potential area for improvement in Yemen. Specific behavioral interventions, such as hygiene training and simulation exercises, could enhance the students' practice.

The lack of significant differences in KAP scores between Sana'a University, the largest university in Yemen, and other universities may reflect a uniformly low baseline of public health education across academic institutions in the country. On the other hand, age and academic year significantly influenced better knowledge and attitude scores, which can be attributed to the cumulative effect of education and experience. Additionally, females had better practices toward the disease, similar to the findings from Saudi Arabia and Bangladesh [16,20]. This can be attributed to women generally adhering to more rigorous hygiene standards compared to men.

Strengths and limitations of the study

When healthcare workers, including medical students, are dealing with an endemic disease, awareness is a cornerstone in managing the disease, limiting its spread, and controlling outbreaks. Additionally, through doctor-patient interactions, healthcare professionals can actively increase public awareness and promote preventive measures. To our knowledge, this is the first KAP study regarding cholera among medical students or healthcare workers in Yemen, with the use of three validated scales, revealing knowledge gaps and shedding light on unsafe practices. However, our study was subjected to selection and response bias because the convenience sampling method used and the ability to distribute the survey online through social media channels led to unequal geographical and demographic variations among the students, limiting the generalizability and representativeness of the findings. The complex nature of some questions (with multiple answers) was possibly challenging as well; participants needed to choose all correct answers to obtain the point allocated for a question, which required deep knowledge of the tested topic. Future interventional and longitudinal studies among a larger population and healthcare personnel are recommended.

## Conclusions

Cholera is a major health concern in Yemen, and our study investigated the KAP toward this disease among medical students in Yemen. Although the students in our sample had moderate levels of knowledge about the disease, there was a major gap between their knowledge and positive attitudes toward practices. Further research among a wider range of students and healthcare workers is recommended to explore the factors contributing to this gap and ways to minimize it. Educational initiatives to raise awareness among students about cholera and its risk factors and preventive measures as well as specific behavioral interventions, such as hygiene training and simulation exercises, which could enhance the students' practice are highly encouraged.

## Appendices

Questionnaire

Part 1: Demographic Characteristics 

**Table 8:** Demographic characteristics

Demographic characteristics	
Age (years)	
Gender	Female
	Male
Marital status	Single
	Married
	Divorced
	Widow/widower
City of residence	Sana'a
	Amran
	Dhamar
	Taizz
	Ibb
	Others
Academic year	1st
	2nd
	3rd
	4th
	5th
	6th

Part 2: Knowledge About Cholera

**Table 9:** Knowledge about cholera *: correct answer; **: full points required selecting all correct options; partial answers scored 0

This section evaluates the knowledge of participants regarding cholera and its causes, transmission, symptoms, and prevention	
What type of infectious agent causes cholera?	Bacteria*
	Virus
	Parasite
	Bug/insect
	I don't know
Through which routes does the cholera pathogen enter the human body?	Ingestion/oral*
	Inhalation/breathing
	Touch/skin
	Blood
	I don't know
What are the modes of transmission for cholera?	Contaminated water*
	Unwashed vegetables and fruits*
	Undercooked seafood*
	Exposed cooked food*
	I don't know
What are the clinical manifestations of cholera infection?	Watery diarrhea*
	Stomachache*
	Nausea and vomiting*
	Muscle cramps*
	Dehydration*
	I don't know
How would you classify the severity of symptoms in cholera cases?**	No symptoms (asymptomatic)*
	Mild*
	Medium*
	Severe*
	I don't know
What is the typical incubation period for cholera?	Minutes-hour
	1-10 hours
	0.5-5 days*
	>15 days
	I don't know
What are the risk factors that increase susceptibility to cholera infection?**	Poor sanitary and hygiene*
	Immunodeficiency*
	Low stomach acid*
	Household exposure*
	Type O blood*
	I don't know
What are the potential complications associated with untreated cholera?**	Death*
	Kidney failure*
	Coma*
	Decreased body electrolytes*
	Hypoglycemia*
	I don't know
Which age groups are most vulnerable to cholera infection?	Children only
	Adults only
	Children and adults*
	I don't know
What are the main treatments?	Rehydration and electrolyte replacement*
	Antibiotics
	Rehydration and electrolyte replacement and antibiotics
	I don't know
Is there a vaccine available for cholera?	Yes*
	No
	I don't know
Can cholera be transmitted between humans and other species?	Human to human*
	Animal to human
	Human to animal
	Insects to animals and humans
	I don't know
How is a diagnosis of cholera confirmed?	Blood test
	Urine test
	Stool test*
	I don't know
Which body systems are primarily affected by cholera?	Digestive (GI)*
	Respiratory
	Nervous
	Urinary
	I don't know
What are the preventive measures to reduce the risk of cholera infection?**	Drink clean water (bottled or previously boiled)*
	Wash hands with soap well and frequently*
	Sterilize raw vegetables and fruits before eating*
	Peel vegetables and fruits and eat unpeeled*
	Cook food well and eat while still hot*
	Avoid eating undercooked seafood*
	I don't know

Part 3: Attitudes Toward Cholera 

**Table 10:** Attitudes toward cholera

This section assesses the participants' perception of cholera as a health concern and their level of worry about its effects					
How dangerous do you perceive cholera to be?	Not at all dangerous	Slightly dangerous	Moderately dangerous	Very dangerous	Extremely dangerous
How concerned are you about cholera re-emerging in the country?	Not at all concerned	Slightly concerned	Moderately concerned	Very concerned	Extremely concerned
How worried are you about getting infected with cholera?	Not at all worried	Slightly worried	Moderately worried	Very worried	Extremely worried

Part 4: Practices Toward Cholera  

**Table 11:** Practices toward cholera *: awarded a point for being a good practice (better practice)

This section explores the personal habits and practices of participants that may influence cholera prevention	
How often do you eat out or order takeout in a typical month?	<5*
	5-10
	10-20
	>20
What do you usually do before consuming raw vegetables?	Wash (water only)
	Wash and peel*
	Do not wash it or peel it
What steps do you take before eating fresh fruits?	Wash (water only)
	Wash and peel*
	Do not wash it or peel it
After the newest re-emergence of cholera, you commit to:	Wash fruits and vegetables more thoroughly*
	Wash hands with soap more frequently*
	Pay more attention to sources of drinking water*
	Minimize eating out/takeaway*
	Avoid eating undercooked seafood and sushi*
	Do not eat unpeeled fresh produce*
	Not changing my practices
